# Heritage event as tourist attraction: the case of *Dymarki Swietokrzyskie, Poland*

**DOI:** 10.1007/s10708-021-10407-4

**Published:** 2021-04-06

**Authors:** Waldemar Cudny, Lee Jolliffe, Anna Guz

**Affiliations:** 1 Faculty of Geographical Sciences, Institute of Urban Geography, Tourism Studies and Geoinformation, University of Lodz, Lodz, Poland; 2 Department of Hospitality and Tourism Management, Ulster Universtiy Business School, Ulster University, Coleraine, Northern Ireland UK; 3 Jan Kochanowski University in Kielce, Kielce, Poland

**Keywords:** Heritage, Heritage event, Reenactment event, Ancient iron smelting, Geographical space

## Abstract

While previous research has focused on heritage visitor attractions few studies have examined visitation to and experience of ancient industrial sites as geographical tourist spaces. This article profiles visitation to *Dymarki Swietokrzyskie*, a heritage reenactment of past events and ancient industrial traditions of iron smelting held in the Polish town of Nowa Slupia. Visitor survey analysis showed visitors perceive the event is a significant tourist attraction. It attracts tourists, is an element of the local heritage industry and contributes to the development of tourism services as part of the heritage tourism sector. The event in Nowa Slupia forms a tourist attraction based on heritage used for creating a contemporary heritage event, evoking specific tourist behaviors and therefore bringing economic profits. Creating the heritage tourism experience here was possible due to the influence of the mix of the elements in a heritage tourism behavioral model consisting of: politics, conservation, authenticity, impact management and interpretation. Examination of the event confirmed the validity of this framework for the analysis of heritage site visitation as well as providing practical insights for both site managers and future researchers.

## Introduction

Different elements of heritage are included among tourist attractions because they are of interest to tourists and thus evoke the need to travel to the tourist destination (Cudny, 2017). According to Lew’s (1987, 558) classification of tourist attractions elements of culture, history, and art form one of the types of human-made attractions. Heritage tourism is evoked among others by well-known heritage sites such as famous museums, such as the Louvre Museum in Paris (Timothy & Boyd, 2003), or complexes of historical buildings like old towns districts or post-industrial sites included in the UNESCO World Heritage List (Cudny, 2017; Smith & Luque-Azcona, 2012). Apart from such popular destinations are smaller heritage attractions. While they are not so popular, they also offer interesting heritage experiences for tourists. Based on such smaller heritage-related attractions tourism can also be well developed (Ashworth & Tunbridge, 1999; Kar et al., 2020). Events are among one of the important tourist attractions (Getz, 2012), and the heritage-related types of events presented in this article are a significant part of the event sector igniting tourism (Light, 1996; Timothy & Boyd, 2003). Moreover, heritage and heritage-related events take part in the creation of geographical space, because they form one of its types called the tourist space (see: Aldskogius, 1977; Butler, 1980; Leiper, 1979; Liszewski, 1995; Włodarczyk, 2009).

This paper therefore examines the role of a reenactment or recreation of a historical event in creating an attraction for tourists and in particular for heritage tourists. This is investigated through the case of Dymarki Swietokrzyskie, a historical reenactment event celebrating traditional iron production in Poland where visitors to the 2019 event were surveyed. As a context for the development of the case and a theoretical background relevant literature is reviewed for different heritage discourses as well as their tourism function and role in heritage tourism, and in heritage events in particular. The research aims to confirm if this type of heritage event can be perceived by visitors as a tourist attraction, evoking heritage tourism, and consequently developing the tourism industry in a small host destination. The case is assessed against a model for the development of heritage tourism originally proposed by Timothy and Boyd (2003) and its impact on the tourism space. The result will provide practical insights for both site managers and researchers and note implications in relation to the Covid-19 pandemic.

Informed by the literature review the research questions for the study of *Dymarki Swietokrzyskie* as a heritage tourist attraction included the following:

- How can a heritage resource be transformed into a reenactment event and become a tourist attraction participating in tourism space creation?
- Who visited the event and how did they perceive it amongst the visitor attractions of the host town?
- What was their experience and how did the event contribute to the economy and tourism attractiveness of the town.

## Literature review

### Concepts of heritage

The concept of heritage is a complex phenomenon, delivered and created by different producers with different functions for society, education, and the economy (Cudny, 2017). The understanding of heritage and its definition has changed over time (Ahmad, 2006; Graham & Howard, 2008). A classic and simplistic view is of heritage as cultural traditions and artifacts inherited from the past and passed onto the next generations (Hardy, 1988). Bowes (1989: 36) included in the heritage concept elements of “major historic sites and institutions, but the entire landscape of the region with its geographic base: farms and field patterns, roads, harbors, industrial structures, villages and main streets, commercial establishments and of course, the people themselves and their traditions and economic activities”. According to Carman (2002) current heritage studies not only analyze what heritage is but also investigate the process of its creation and interpretation resulting from contemporary social and political processes.

Another view of heritage exists in heritage studies. It presents heritage as a “value loaded concept, embracing (and often obscuring) differences of interpretation that are dependent on key variables, such as class, gender and locality; and with the concept itself locked into wider framework of dominant and subversive ideologies” (Hardy, 1988, 333). According to Tunbridge and Ashworth (1996) heritage may be approached using different dimensions related to history but also related to identity, power and economy. In such an approach heritage may be a synonym for a relic of the past; a product of modern conditions however with attribution and influence of the past. Moreover, heritage can encompass cultural and artistic products created in the past or present, elements from the natural environment remaining from the past original and typical enough to be passed to the next generations; a major commercial activity attached to the heritage industry based on selling products including a heritage component.

### Heritage tourism

Heritage tourism overlaps with cultural tourism because culture is part of cultural landscapes of the present and the past (Prentice, 1994; Richards, 2001). Some authors state that cultural tourism encompasses both heritage consumption and consumption of the contemporary cultural products at visited destinations (Richards, 2001). Other authors such as Moscardo (2001) saw the distinction between heritage and cultural tourism lying in the focus on the past or present in the latter case (Timothy & Boyd, 2003).

Heritage tourism may be also understood independently (Timothy, 2018; Timothy & Boyd, 2003). Poria et al. (2003) stated that traditional heritage tourism is distinguished on the basis of the site attractions, places or artifacts displayed at a specific place attracting heritage tourists. The authors defined heritage tourism as “a subgroup of tourism, in which the main motivation for visiting a site is based on the place’s heritage characteristics according to the tourists’ perception of their own heritage” (Poria et al., 2003, 247). Timothy (2011, 4) indicated “Heritage tourism refers to travelers seeing or experiencing built heritage, living culture or contemporary arts. Its resources are tangible and intangible and are found in both rural and urban settings”.

Heritage attractions form the basis for the development of heritage tourism and the economic sector known as the heritage industry. According to Lew (1987, 554)”Tourist attractions consist of all those elements of a nonhome place that draw discretionary travelers away from their homes. They usually include landscapes to observe, activities to participate in, and experiences to remember”. Swarbrooke (1994, 222) notes the following groups of heritage attractions: historic buildings and monuments; sites of important past events like battles; traditional landscape and indigenous wildlife; language, literature, music and art; traditional events and folklore practices; traditional lifestyles including food, drink and sport.

### Events as heritage attractions

Amongst the important heritage attractions evoking heritage tourism are special events (Chhabra et al., 2002; del Barrio et al., 2012; Prentice & Andersen, 2003; Xie, 2004) Their organization may help in promotion, creation and interpretation of heritage (Timothy & Boyd, 2003). Previous studies have shown that special events such as historical reenactments can encourage visitors to spend longer at a historic site (Light, 1996). Special events organization can also trigger public awareness of historic values and heritage while at the same time creating economic educational and socio-cultural benefits (Janiskee, 1996).

Getz wrote (2012, 37) “Events, by definition, have a beginning and an end. They are temporal phenomena, with planned events the event program or schedule is generally planned in detail and well publicized in advance. Planned events are also usually confined to particular places, although the space involved might be a specific facility, a very large open space, or many locations”.

Among planned events are reenactment events focused on reflection and remembrance of historical occurrences and their relationship to today. Such events could be organized on anniversary dates during significant periods of time and their organization is similar to special events or festivals (Frost & Laing, 2013). Reenactment is associated with the remembrance of important historical events (wars, battles for example), and with living history where performances recreate life of past generations by simulating ways of life (modes of dress, recreation, work, transportation, speech) (Agnew et al., 2020; Yamamura, 2018). Agnew (2007, 299) stated that “Recent scholars use the term to include everything from living history museums, technical reconstructions and 'nostalgia' toys (e.g. tin figures, dioramas and architectural models) to literature, film, photography, video games, television shows, pageants, parades and, reenactment's most ubiquitous instantiation, social and cyber groups devoted to historical performance. What these forms share is a concern with personal experience, social relations and everyday life, and with conjectural and provisional interpretations of the past”.

Reenactment events are often both cultural heritage events and living history performances. Such events focus on presenting past events representing and re-living old traditions, religious practices, military, political, historical, economic and other past events. These events habitually encompass fantasy role play by their participants. Reenactment events are organized by different stakeholders, however mostly by private persons or associations interested in the recognition, remembrance and popularization of the past and heritage connected with history (Daugbjerg et al., 2014; McCalman & Pickering, 2010).

According to Timothy and Boyd (2003, 224) interactivity is central in heritage visitation. A site offering possibility to participate in reenactment events, historic activities, to handle and use historical artifacts is much more interesting for visitors than a place which offers only motionless displays and historic photographs. Timothy and Boyd (2003, 225) point out that heritage visitors may be attracted with the use by places of the world’s industrial past (e.g. factories, places of natural resources extraction) as heritage attractions offering direct interactive experience with historic industrial activities.

A behavioral model of heritage tourism was presented by Timothy and Boyd (2003, 7–9). According to the model heritage exists in two types of environments. The first type is the phenomenal environment encompassing natural and cultural elements objectively present in the space. The second type is behavioral environment where the social and cultural facts are passed through a filter of human values. As a result of this filtration actions are created which may transform the heritage from the phenomenal environment into the behavioral one. In this way the heritage objectively present in the environment may be noticed, interpreted and valued and may become part of behavioral environment and a resource for tourism development.

Heritage is something that is perceived as value and what we want to keep and pass to next generations. Not everything from the phenomenal environment is selected as heritage worth passing to the future. That which is selected goes through a number of perception filters. First it needs to be perceived as heritage to move from the phenomenal to the behavioral environment. Second it needs to be valued as a commodity which is worth marketing and can be sold to consumers such as tourists. If it is worthwhile then it passes through an economic filter, gains economic function and becomes a part of heritage industry (Timothy & Boyd, 2003, 7–9).

Central to the process is the heritage tourism experience which is the most important result for visitors taking part in the heritage tourism. Experiences understood as extraordinary, life-enriching happenings that the visitors may participate in are vital for contemporary tourist attractions (Cudny, 2017; Cudny & Jolliffe, 2019). The heritage tourism experience is influenced by a mix of different elements (Timothy & Boyd, 2003, 7–9): supply and demand; the nature of heritage landscape that has been conserved and protected; the impact heritage creates and leaves within destinations; the management of heritage attractions and its authenticity; interpretation and presentation of heritage; politics and the role it plays in forming of heritage experience.

Moreover, heritage tourism is responsible for tourism space creation. Tourism space is part of geographical space which according to Thrift (2003, 95) “Is not a commonsense external background to human and social action. Rather, it is the outcome of a series of highly problematic temporary settlements that divide and connect things up into different kinds of collectives which are slowly provided with the means which render them durable and sustainable”. Cudny (2014, 138) based on Thrift (2003) distinguished four dimensions of geographical space i.e. the first space of empirical constructions (visible, measurable, and physically present), the second so-called unblocking space of connections (social and economic) through which the world interacts, the third space called the image space encompassing pictures and other images with space representations, and the fourth space called place space which “… consists of particular rhythms of being that confirm and naturalize the existence of certain spaces…places not only offer resources of many different kinds … but they also provide cues to memory and behavior”.

According to Włodarczyk (2009, 75) “Tourism space is the part of the geographical space in which the phenomenon of tourism occurs. The necessary and sufficient condition for classifying a part of geographical space as tourism space is tourism, regardless of its size and nature”. Because the tourism space is part of geographical space it includes the same four dimensions identified by Thrift (2003) as the geographical space itself.

## Methodology

Taking a mixed method approach the research methods used to address the research questions included participant observation and a semi-structured questionnaire-based survey. The researchers also collected information on the event from literature and the event organizers. The choice of the event was one of convenience, as the authors had access to the event.

Participant observation involves purposeful and planned participation in the observed phenomenon or event. This method aims to establish the course of the event as well as the behaviors of its organizers and spectators (Veal, 2006). The questionnaire-based survey is commonly used in the social, economic and behavioral sciences. This involves collecting opinions from human respondents with the use of a standardized questionnaire, using a list of questions devoted to a specified research problem which the research seeks to answer (Saris & Gallhofer, 2014). Such a survey may be used to obtain quantified information about respondents’ opinions, behavior and attitudes towards specified phenomena (Veal, 2006). The most popular types of questionnaires include structured and semi-structured ones, the latter was used in the research. The semi-structured questionnaire includes a list of predefined closed questions (with answer options to choose from) supplemented with open-ended questions to be filled in by the respondents (Simon, 2006). The different types of questionnaire-based surveys include an intercept survey. This is a research technique used to acquire on-site opinions from an audience visiting tourist destinations. This type of survey is often used during tourist events (Kaplanidou et al., 2013; Schneider & Backman, 1996; Shani et al., 2009). It involves intercepting available respondents on site or during an event (Veal, 2006).

Participant observation was conducted on both days of the *Dymarki Swietokrzyskie* event. The researchers observed the behavior of the organizers, volunteers and visitors; observation was documented with notes and photographs. A questionnaire-based visitor survey was conducted on both days of the event, as an intercept (self-reported) survey. Questionnaires were distributed by five trained researchers to every fifth individual entering the event. The authors distributed 300 questionnaires. Out of 163 collected questionnaires, 135 correctly completed ones were analyzed.

Data obtained from a questionnaire-based survey may be analyzed in several ways, including descriptive analysis adopted in this article, using frequencies and mean values representing the respondents' opinions and behaviors (Veal, 2006). The subsequent examination of the survey results employed the heritage tourism behavioral framework initially developed by Timothy & Boyd (2003). This includes testing this behavioral model and examining its relation to tourist space.

## Case study

**Fig. 1:**
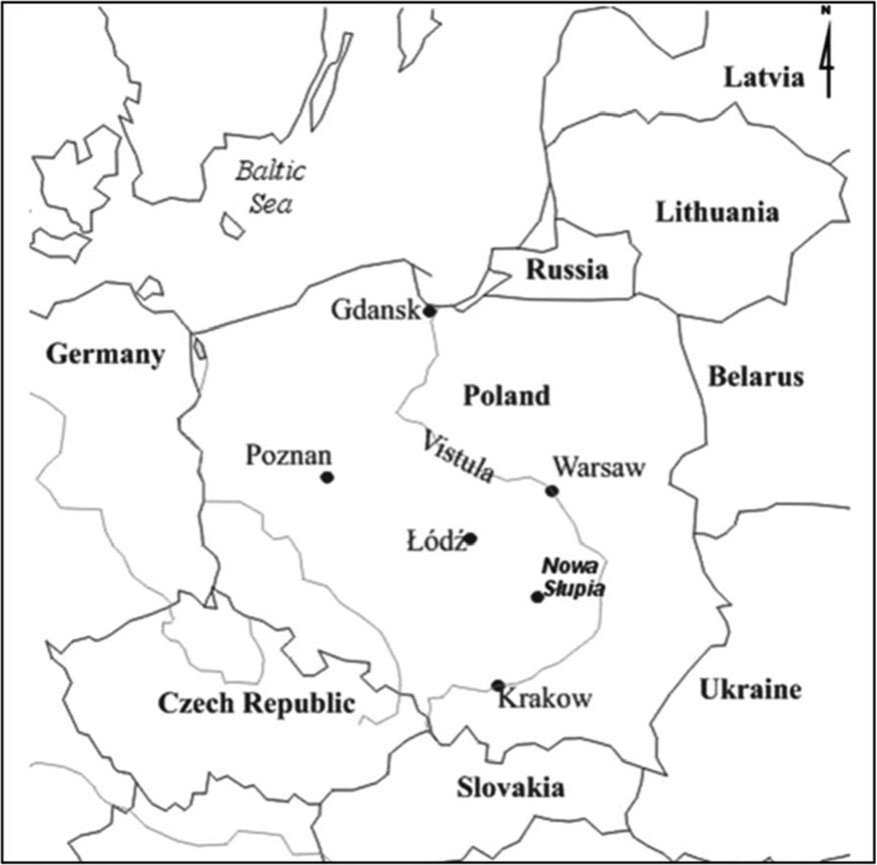
Location of Nowa Słupia on the map of Poland. *Source*: Authors

*The Dymarki Swietokrzyskie* event is held in the town of Nowa Slupia in Swietokrzyskie Province, an administrative region located in east-central Poland, in the area of the Swietokrzyskie Mountains (Fig. 1). Nowa Slupia, a small destination inhabited by about 1400 people, regained its municipal status in 2019. The town possesses several tourist assets, such as the Museum of Ancient Metallurgy, the Cultural-Archaeological Centre, St Lawrence Church, the Abbot's House or the historic figure of the Stone Pilgrim. An important attraction in the town is the annual event, known as *Dymarki Swietokrzyskie*. In the close vicinity of Nowa Slupia is Swietokrzyski National Park and a historical pilgrimage centre—the Holy Cross Abbey. Nowa Slupia became known in the twentieth century as a center of research on ancient metallurgy, reflecting a history of production.

About 2000 years ago, during the Roman period, the territory of the Swietokrzyskie Mountains was the largest metallurgic district in Central Europe. Between the first century BC to about fourth century AD, iron was smelt there, using an ancient method, in charcoal furnaces, called bloomeries (*dymarki*). The furnaces were single-use clay structures, in which the smelting process took place. After it was completed, the furnaces were dismantled and a hot mass of iron was taken from the inside. Nowadays, there are 1700 known places in the Świętokrzyskie region where such furnaces were used, including those found around Nowa Slupia.

During the first four centuries after Christ, about 8000 tons of iron was produced in the region, probably for nearby tribes and possibly distributed all over Central Europe and in Roman provinces. There is abundant evidence of trade contacts between the local population, representing the Przeworsk culture, and the Roman provinces. Artefacts include Roman coins found during archaeological excavations, ceramics and glass products (Orzechowski, 2002).

Early findings regarding metallurgy in the Swietokrzyski region date back to the beginning of twentieth century. Archaeological studies accelerated in the 1950s. In 1958, the area of archaeological excavations, with relics of antique furnaces on the Nowa Slupia terrain, was roofed. This was the beginning of the Museum of Ancient Swietokrzyskie Metallurgy. Similar sites were found in other areas of the Swietokrzyski region, but it was Nowa Slupia that became a symbol of ancient metallurgy, partly due to organizing *Dymarki Swietokrzyskie,* a large event presenting the heritage of this industry (Świętokrzyskie region, n.d.).

The first public demonstration of smelting iron in bloomeries took place in Nowa Slupia, in 1967. Local social activists and the members of the Polish Tourism and Sightseeing Society took advantage of the local archaeological discoveries to create a significant tourism event for the region. They wished to save the cultural heritage related to ancient metallurgy from oblivion by creating an event which would be an attraction enhancing local tourism. The initiative was well received and is currently known as *Dymarki Swietokrzyskie*. In the 1967 World Year of Tourism Polish authorities announced a competition for the most interesting tourist event which was won by the event (Bielenin 1992).

In subsequent years, interest in the event has been growing. Its main attraction is still the detailed reconstruction of the iron smelting process in traditional clay furnaces, based on historical and archaeological knowledge. In the 1990s the program included a number of additional events, such as presentations and reenactment of the daily lives of the ancient inhabitants of the Swietokrzyski region, and presentations of the Roman legionnaires' outfits, weaponry and everyday living conditions, Roman cuisine, craftsmanship and forms of entertainment. There are presentations of the everyday life of some barbarian tribes that lived outside the Roman Empire (Celts, Goths, Dacians, the Harii, the Silings). The event includes reconstructions of the battles between barbarians and Romans. Visitors can experience presentations of contemporary folklore from the Swietokrzyski region, a handicrafts fair, or music concerts. Several hundred presenters and reconstruction organizers take part in the event. They arrive from Poland, Belarus, the Czech Republic, Romania, Germany, Russia, Ukraine and Denmark. The overall number of the visitors attending all the editions organized so far has exceeded one million (Dymarki Świętokrzyskie, n.d.)

In the organizers' opinion, *Dymarki Swietokrzyskie* has become a mass event, aiming to preserve and disseminate heritage related to ancient iron smelting. The event is a major attraction for tourists interested in heritage increasing the revenues of the local gastronomic and hospitality sector. Today, *Dymarki Swietokrzyskie* is the largest event of this type in the region and one of the largest in Poland. Organizers report that the number of tickets sold for the 2019 edition was 19,793 compared to 2018 with 22,295 and 2017 with 21,201. The number of presenters in the 2019 was 120 who took part in reenactment and historical presentations. They were mostly coming from Poland, Czech Republic, and Ukraine. The same number of participants was recorded in the previous 2019 edition. However, in 2020, during the pandemic year *Dymarki Swietokrzyskie* was limited the number of presenters dropped to 50. The main organizer of the event is the Municipal Centre of Culture, Sport and Tourism (Gminny Ośrodek Kultury Sportu i Turystyki—GOKSiT) in Rudki. It is a self-governing cultural institution whose main aim is to satisfy the cultural needs of the inhabitants of the municipality (*gmina)* of Nowa Slupia.

The event is financed from ticket sales, fees for rental sales and food stands, and sponsorships. It is organized by six employees of GOKSiT. One week before the event, more people from the Municipal Hall in Nowa Slupia are engaged, as well as a number of volunteers. According to the Polish law, GOKSiT is obliged to provide security measures and medical care for the duration of the event. In 2019, the event was guarded by 60 employees of a specialist security firm.

Main *Dymarki Swietokrzyskie* events take place on the premises of the Cultural-Archaeological Centre (Centrum Kulturowo-Archeologiczne—CKA) in Nowa Slupia. It is an educational-archaeological park, administered by the municipality. Its purpose includes researching cultural heritage and organizing shows related to ancient iron smelting. CKA was established in 2011, with the help of the European Regional Development Fund. The project launched during the 45th edition of the event in 2011 was worth around 900,000 Euro.

CKA covers about 4 hectares and its facilities were created based on the archaeological excavations from the times of the Roman period, conducted in different parts of Poland. It is one of the few places in Europe presenting most aspects of ancient iron metallurgy. The architectonic conception is modelled on the Barbarian and Roman building styles. The CKA area comprises 11 sites, reconstructed on the basis of archaeological excavations and prepared using traditional building methods from 2000 years ago. This includes: pit-houses and huts where people lived, workshops, the long house, iron mine, sauna, bloomeries and a pottery furnace site, a fragment of a Roman camp with reconstructed Hadrian's Wall and guards' tower, and a holy orchard (devoted to the spirits of nature). At CKA events and thematic educational meetings are organized, presenting different aspects of life of the inhabitants of Europe and the Swietokrzyski region during Roman times. The centre is a popular tourist attraction, visited by 40,772 people in 2019.

## Visitor survey and participant observation

**Fig. 2:**
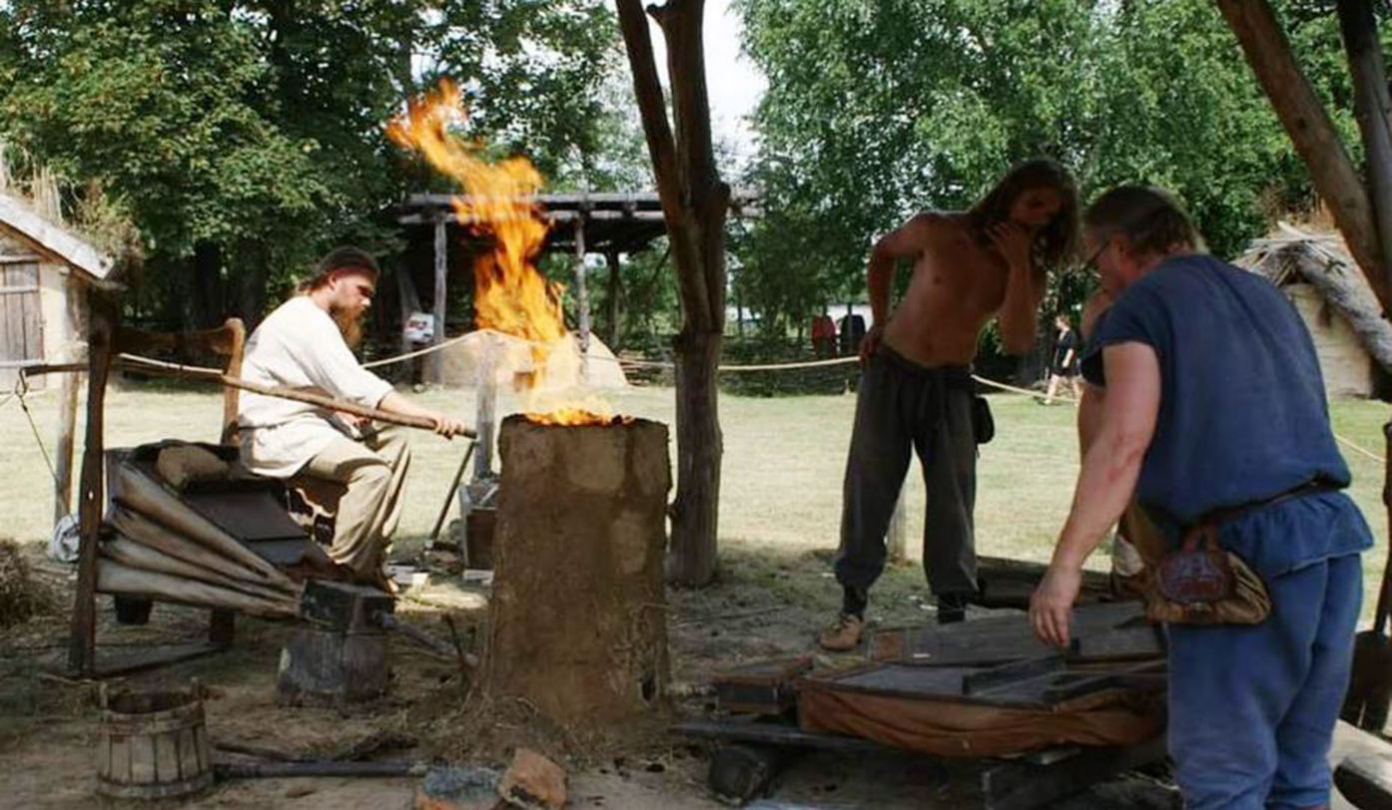
Bloomeries demonstration during the *Dymarki Świętokrzyskie* event. *Source*: Authors

This was conducted during the 53rd edition of *Dymarki Swietokrzyskie*, August 17–18. 2019. On the first day (August17th), the "Iron Circle Archaeological Festival" was officially started. The festival, the main event of *Dymarki Swietokrzyskie* was held at the Cultural-Archaeological Centre in Nowa Slupia (CKA). The festival included several shows, presenting various aspects of life in the ancient Barbarian world and in the area of Roman provinces. The main attraction was specialist presentations dealing with the reconstruction of antique blacksmithing, gilding, bronzing and other crafts, arranged on an area where ancient smelting furnaces had been reconstructed (*piecowisko*) (Fig. 2). In the afternoon, the visitors could view a reenactment presenting the reconstruction of the full iron smelting cycle, using the 2000-year-old technology.

**Fig. 3:**
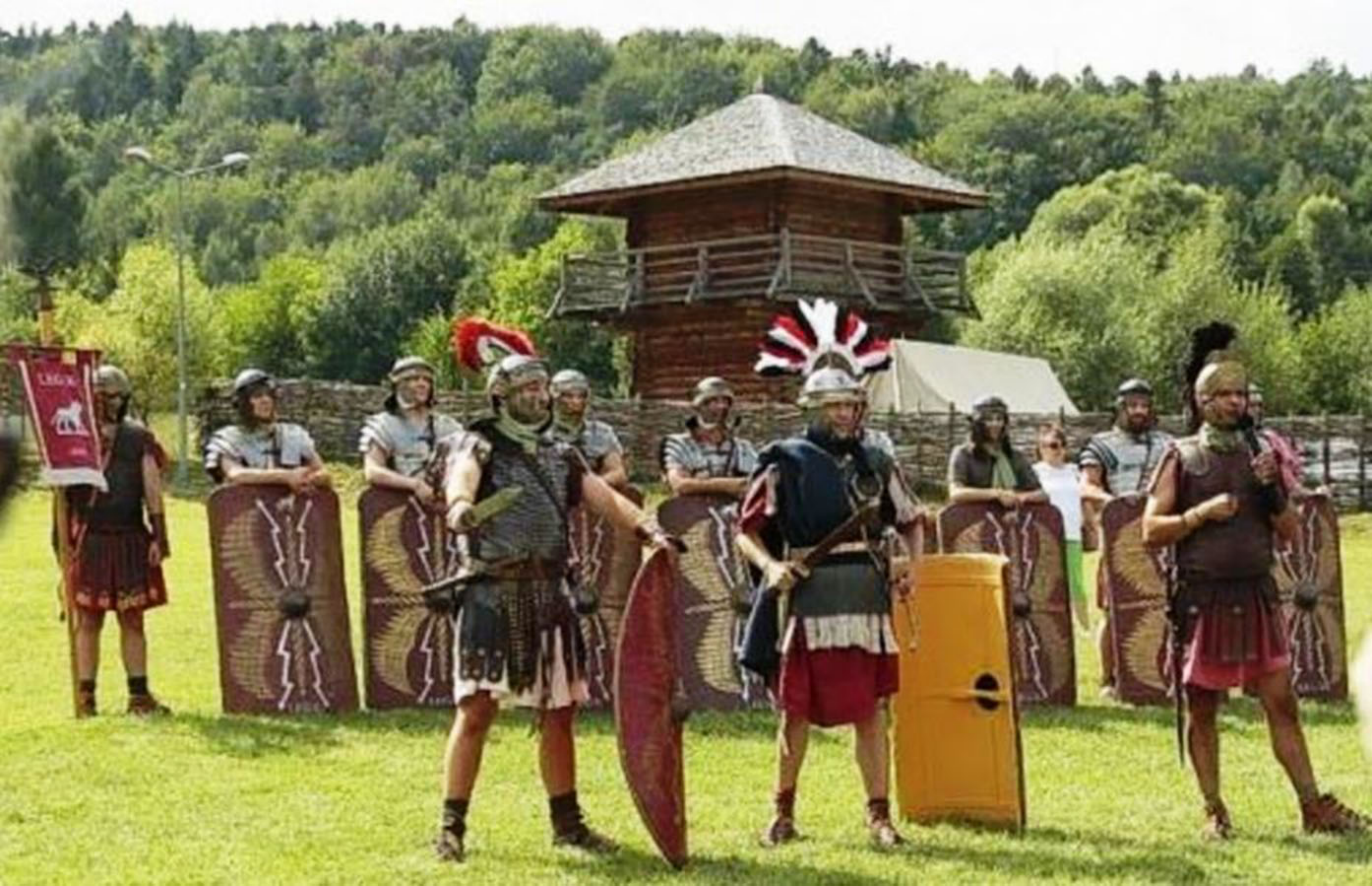
Presentation of Roman weaponry during the *Dymarki Świętokrzyskie* event. *Source*: Authors

The shows also included presentations of Romans' and Barbarians' outfits and weapons, reconstructions of the daily life from the Roman times (Fig. 3), as well as of the battles fought by Barbarian tribes (Germans, Balts, Dacians, Sarmatians) with the Romans. In another zone, situated outside CKA, visitors could enjoy the Swietokrzyski Festival of Folk Culture, watching performances by regional bands and singers, and meeting contemporary folk artists, presenting their paintings and sculptures. The festival aims to preserve and propagate the folk culture of the Swietokrzyski region.

The first day of *Dymarki Swietokrzyskie* ended with an evening concert on the Stage by the Forest. It featured many music ensembles, presenting different music styles. Moreover, throughout the event, on the premises of CKA and the adjacent streets, the tourists could visit an artistic handicraft fair.

On the second day of *Dymarki Swietokrzyskie*, the "Iron Circle Archaeological Festival" and the Festival of Folk Culture was continued as well. Apart from that, the results of the painting and sculpting competitions were announced and the winning folk musicians performed. It was possible to taste local dishes, attracting crowds of tourists and inhabitants of Nowa Slupia.

The visitor survey was conducted on both days of *Dymarki Swietokrzyskie*, with questionnaires distributed to every fifth individual entering the event. From the 163 collected questionnaires, 135 correctly completed ones were analyzed. Respondents who had attended *Dymarki Swietokrzyskie* (91.9%) were included in the group of repeat visitors. The majority of 113 (83.7%) were tourists from outside the town, 22 people (16.3%) were inhabitants of Nowa Slupia. Respondents included 80 women (59.3%) and 55 men (40.7%). The largest age group included respondents aged 36–45 (23%). It was followed by groups aged 19–25 (20%) and 46–55 (17%). Regarding the education level, the predominant group included people with university (34.8%) and secondary (31.1%) education (Table 1).

**Table 1:** Respondents' age and education structure

	Number	%
*Age*		
15–18 years	15	11.1
19–25 years	27	20.0
26–35 years	20	14.8
36–45 years	31	23.0
46–55 years	23	17.0
56–65 years	14	10.4
66 years and over	5	3.7
Total	135	100.0
*Education*		
Primary	2	1.5
Secondary Junior	12	8.9
Vocational	21	15.6
Secondary Senior	42	31.1
Post-Secondary	3	2.2
Higher (University)	47	34.8
Not given	8	5.9
Total	135	100.0

The largest group (74.4%) of respondents came from Swietokrzyskie Province, which Nowa Slupia belongs to. Of these visitors 16.3% were town residents and 58.1% came from the province, but from outside the town. The latter group can be treated as regional visitors. On the other hand, 25.6% of the respondents came from outside Swietokrzyskie Province and those can be classified as domestic visitors from beyond the region.

## Research results

The survey included questions regarding seven main issues: the character of the visit at *Dymarki Swietokrzyskie*, the indication of the main tourist attraction of Nowa Slupia, the uniqueness of the experience related to attending the event, the impact of *Dymarki Swietokrzyskie* on various elements of the town's tourist attractiveness and their connection with the local cultural heritage, event evaluation and willingness to revisit the event.

In the first question, respondents were asked to choose the main element characterizing their visit. They were to define their place of residence and state whether the event was their main reason to come to Nowa Slupia. A majority of 65.2% indicated that they came from outside Nowa Slupia and had arrived specially for the event. Definitely fewer respondents (12.6%) were from outside Nowa Slupia and had arrived to visit the town, attending the event coincidence. A smaller number (16.3%) identified as town residents, participating in the event in their free time. Thus, a great majority of the respondents had come specially for the event, which indicates that it is a significant attraction drawing visitors from outside of the destination.

Respondents arriving from outside Nowa Slupia (113) were asked additional questions about who they had come with and whether that had spent the night in the town. Of these respondents 53.1% travelled with their friends, 33.6% with family and 6.2% with their colleagues from work. Only 7.1% of the respondents from outside the town arrived alone. As many as 85.8% did not stay overnight in Nowa Slupia. The small percentage of those who did may result from their place of residence as 74.4% of all respondents came from Swietokrzyskie Province, so they could easily travel to the event and return home on the same day. The fact that most respondents (92.9%) travelled in somebody's company raises the status of the event due to a larger number of visitors. On the other hand, that only 14.2% of them spent the night in the town reduces the economic impact, because the fewer overnight stays, the shorter the stay and the smaller the economic impact for the host destination.

Respondents were to indicate the main tourist attraction in Nowa Slupia in a closed question, listing major local tourist attractions:

- (a) Museum of Ancient Metallurgy in Nowa Slupia
- (b) Cultural-Archaeological Centre
- (c) St Lawrence Church
- (d) *Dymarki Swietokrzyskie*
- (e) The Abbot's House
- (f) The Stone Pilgrim

According to 65.2% of respondents, *Dymarki Swietokrzyskie* is the greatest tourist attraction of the town. In the residents' opinion, the main attraction is St Lawrence Church (27.3%), followed by *Dymarki Swietokrzyskie* (22.7%). Most tourists believed the latter was most attractive, tourism-wise (73.5%).

The next question was: "Is visiting *Dymarki Swietokrzyskie* a unique experience to you, increasing the tourist attractiveness of Nowa Slupia?" As indicated by Cudny (2016), modern societies are oriented towards cultural asset consumption, which are unique experiences to them, giving them a chance to escape the everyday routine. Events, such as festivals, offer excitement, an opportunity to flee the boredom of everydayness (Scott, 1995), due to their otherness, uniqueness. As many as 44.5% of the respondents answered "*yes"* to this question (50% of the residents and 44.4% of tourists), while the answer *"definitely yes"* was chosen by 37.8% (31.8% of residents and 38.9% of tourists). Other variants ("*difficult to say", "no"," definitely no"*) appeared in 17.7% of all answers.

The next question presented a table containing statements regarding the influence of *Dymarki Swietokrzyskie* on the tourist attractiveness of Nowa Slupia (Table 2). Cracolici and Nijkamp (2009) note tourist attractiveness is a complex construct encompassing all tourist attractions offered to tourists, as well as the tourist services available at the visited place. The broadly understood concept of tourist attractiveness consists of elements like attractions, tourist services, tourists’ satisfaction with stay. The respondents were therefore asked to say whether *Dymarki Swietokrzyskie* is a tourist attraction of the town, whether it enhances tourism in the town and increases the revenues of the enterprises offering tourist services (e.g. hotels, restaurants). Moreover, respondents were to express their opinions on the connection between the event and the local cultural heritage. With this question, the five-grade Likert scale was used, respondents could choose one of the following answers when referring to each statement: *I definitely disagree, I rather disagree, I don't know, I rather agree, I definitely agree.*

**Table 2:** Evaluation of *Dymarki Swietokrzyskie* as a tourist attraction

Answer variant	Total		Residents		Tourists	
	Number	%	Number	%	Number	%
*Dymarki Swietokrzyskie can be considered a very important attraction drawing tourists to the town*						
I definitely disagree	2	1.5	0	0.0	2	1.8
I rather disagree	6	4.4	0	0.0	5	4.4
I don't know	3	2.2	1	4.5	3	2.7
I rather agree	45	33.4	7	31.8	38	33.6
I definitely agree	79	58.5	14	63.6	65	57.5
Total	135	100	22	100.0	113	100.0
*Dymarki Swietokrzyskie increases the number of tourists in the town*						
I definitely disagree	1	0.7	0	0.0	1	0.9
I rather disagree	6	4.4	1	4.5	6	5.3
I don't know	6	4.4	1	4.5	5	4.4
I rather agree	36	26.7	6	27.3	29	25.7
I definitely agree	86	63.8	14	63.6	72	63.7
Total	135	100	22	100.0	113	100.0
*Dymarki Swietokrzyskie causes an increase in the revenues of the enterprises offering tourist services in the town (e.g. hotels, restaurants, shops)*						
I definitely disagree	2	1.5	0	0.0	2	1.8
I rather disagree	2	1.5	0	0.0	2	1.8
I don't know	11	8.1	0	0.0	11	9.7
I rather agree	40	29.6	9	40.9	31	27.4
I definitely agree	80	59.3	13	59.1	67	59.3
Total	135	100	22	100.0	113	100.0
*Dymarki Swietokrzyskie is strictly connected with the local cultural heritage related to ancient iron smelting*						
I definitely disagree	0	0	0	0.0	0	0
I rather disagree	2	1.5	1	4.5	1	0.9
I don't know	8	5.9	0	0.0	8	7.1
I rather agree	43	31.9	7	31.8	36	31.9
I definitely agree	82	60.7	14	63.6	68	60.2
Total	135	100	22	100.0	113	100.0

The first statement in the table was: *Dymarki Swietokrzyskie can be considered a very important attraction drawing tourists to the town*. More than half of the respondents (58.5%) said *I definitely agree.* Both, the residents and the tourists chose this answer most often (Table 2). The next statement was: *Dymarki Swietokrzyskie increases the number of tourists in the town.* As many as 90.5% of all respondents confirmed (63.8%—*I definitely agree*, 26.7%—*I rather agree*). A similarly high percentage of indications appeared among residents and tourists. The structure of answers shows that *Dymarki Swietokrzyskie* is an event attracting tourists so their increased number in the town is noticeable. The following statement was: *Dymarki Swietokrzyskie causes an increase in the revenues of the enterprises offering tourist services in the town (e.g. hotels, restaurants, shops).* As many as 88.9% of the answers were positive (59.3%—*I definitely agree* and 29.6%—*I rather agree*). This statement was confirmed by residents and tourists alike, indicating a positive impact of the event on the development of tourism industry.

For the next statement: *Dymarki Swietokrzyskie is strictly connected with the local cultural heritage related to ancient iron smelting,* well over half of the respondents (60.7%) definitely agreed with this statement: 63.6% of Nowa Slupia inhabitants and 60.2% of the tourists. The answers clearly indicate that in the respondents' opinion, *Dymarki Swietokrzyskie* is a tourist attraction of the town, enhancing tourism and increasing the revenues of the enterprises offering tourist services. Respondents believe the event is strongly related to local cultural heritage, based on ancient iron smelting.

Next, respondents were to evaluate individual elements of Dymarki Swietokrzyskie by allocating a number of points to individual elements, from 1 (lowest rating) to 5 (highest rating) (Table 3). The points were allocated for: event organization, event venue, information available during the event, safety, sanitary facilities, artistic setting, sales booths organization. Most elements were rated high, at over 4 points. The highest rated ones were the organization of stands selling handcraft products (average 4.5), event venue (4.4), safety during the event (4.4) and artistic setting (4.4). The lowest rated element was the sanitary facilities (average 3.5), due to the insufficient number of toilets. High ratings indicate a considerable level of satisfaction with event visitation. Satisfaction is an element related to positive feelings towards the visited event, encouraging the visitors to participate in the event again (Christou et al., 2018; Kim et al., 2010).

**Table 3:** Rating individual elements of *Dymarki Swietokrzyskie*

Rate the elements of the event, with points from 1 (lowest rating) to 5 (highest rating)	Average rating		
	Total	Residents	Tourists
Event organization	4.2	4.2	4.2
Event venue	4.4	4.9	4.3
Information during the event	3.8	4.0	3.8
Safety during the event	4.4	4.6	4.4
Sanitary facilities	3.5	3.3	3.5
Artistic setting	4.4	4.4	4.4
Organization of sales stands	4.5	4.6	4.5

To review the opinions about the event in more detail respondents were asked to evaluate *Dymarki Swietokrzyskie* in one sentence (Table 4). The answers were divided into groups of similar content. The majority of the answers were positive. Nearly half of all respondents (49.7%) evaluated the event as "excellent" or "very good" and 41.5%—as "good". Half of the residents described it as "good " and 51.3% of the tourists—as "excellent" or "very good".

**Table 4:** One sentence evaluations of the event

Evaluate the event in one sentence	Total		Residents		Tourists	
	Number	%	Number	(%)	Number	(%)
An excellent, very good event	67	49.7	9	40.9	58	51.3
A good event	56	41.5	11	50.0	45	39.8
No opinion	6	4.4	2	9.1	4	3.5
Event evaluated negatively	6	4.4	0	0.0	6	5.3
Total	135	100	22	100.0	113	100.0

In addition, respondents were asked whether they intended to take part in the next *Dymarki Swietokrzyskie*. Slightly over half of all the respondents answered *yes* (51.1%) and 36.3% said *rather yes*. Both, residents and tourists gave mostly positive answers (Table 5). The structure of answers to this question seems to be linked to the highly positive evaluation of *Dymarki Swietokrzyskie*, presented above. Most respondents who praised the event intended to revisit it. The connection between positive opinions and the willingness to revisit an event has also been confirmed by other authors (Cole & Chancellor, 2009; Son & Lee, 2011).

**Table 5:** Willingness to revisit *Dymarki Swietokrzyskie*

Do you intend to take part in the next *Dymarki Swietokrzyskie*?	Total	Residents	Tourists			
	Number	%	Number	Number	%	Number
Yes	69	51.1	16	72.7	53	46.9
Rather yes	49	36.3	6	27.3	43	38.1
Rather no	4	3.0	0	0.0	4	3.5
No	2	1.5	0	0.0	2	1.8
I don't know	11	8.1	0	0.0	11	9.7
Total	135	100.0	22	100.0	113	100.0

## Discussion

Heritage is the outcome of the historical development of a given area, becoming heritage only when some elements of the past are chosen as worth preserving and passing on to the next generations (Turnbridge & Ashworth, 1996; Waterton et al., 2017). Dymarki Swietokrzyskie represents such a case. The core element of the event are attractions related to the heritage of ancient metallurgy. The main attraction is the reenactment of iron smelting, using a 2000-year-old method. Supplementary events present related history and contemporary folklore.

In the mid-1950s, a group of archaeologists and historians conducted intensive research on the relics of ancient metallurgy around Nowa Slupia. As a result, they identified that this aspect of the ancient history of the region was worth displaying and preserving for the future generations. Following this idea, the Museum of Ancient Swietokrzyskie Metallurgy was established and *Dymarki Swietokrzyskie* was created.

The event may be treated as a part of heritage defined by Hardy (1988) and Bowes (1989). It represents the features of a reenactment heritage event (Frost & Laing, 2013; McCalman & Pickering, 2010) involving a reconstruction of past events, behaviors and heritage activities by activists and heritage organizations. Today, the event is a major commercial attraction, co-organized by the local authorities in order to preserve the ancient metallurgy heritage. As indicated by scholars (Turnbridge & Ashworth, 1996; Timothy & Boyd, 2003), attractions of this type enhance the development of the local heritage and tourism industry based on selling products with a heritage component.

Heritage events like *Dymarki Świętokrzyskie* stimulate both event and heritage tourism (Backman et al., 1995; Getz, 2012; Park, 2013; Timothy, 2011; Timothy & Boyd, 2003). The most important part of *Dymarki Swietokrzyskie* is the reenactment of ancient iron smelting, thus creating a unique and extraordinary experience that is valued by the event participants. This is in line with previous research (McCalman & Pickering, 2010; Timothy & Boyd, 2003), that emphasized the role of events offering unique reenactment performances and experiences in the creation of heritage tourist attractions.

The significant role of *Dymarki Swietokrzyskie* in tourism development is confirmed by attendance data (19,793 in 2019, over a million participating in all editions), as well as the structure of the answers obtained during questionnaire-based survey. Thus, *Dymarki Swietokrzyskie* is a tourist attraction increasing tourism and tourist attractiveness, as well as an element of heritage industry, supporting local tourist service enterprises. An element created for the purposes of the event is the Cultural Archaeological Centre in Nowa Slupia, opened in 2011. It runs educational activity related to the regional heritage of ancient metallurgy and hosts *Dymarki Swietokrzyskie* every year. The considerable impact of the event on tourism development confirms earlier studies on the role of events as heritage attractions (Swarbrooke, 1994; Chhabra et al., 2002; Prentice & Andersen, 2003; Xie, 2004; McCalman & Pickering, 2010; del Barrio et al., 2012).

**Fig. 4:**
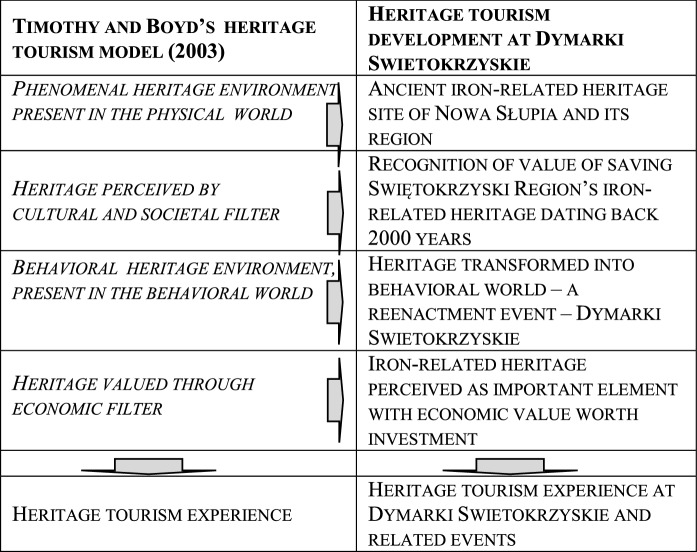
Heritage tourism development stimulated by *Dymarki Swietokrzyskie*. *Source*: Own elaboration on the basis of Timothy and Boyd (2003)

Based on the framework of heritage tourism (Timothy & Boyd, 2003), we illustrated heritage tourism development stimulated by *Dymarki Swietokrzyskie* (Fig. 4). Firstly, it is an authentic event, based on archaeological findings (artefacts, historical analyses) concerning ancient iron smelting in the Swietokrzyski region 2000 years ago (about 1700 sites with artefacts were discovered in the Świętokzyskie Region). Consequently, ancient metallurgy can be classified as a phenomenal heritage environment, representing the physical world and encompassing natural and cultural elements, objectively present in the space and created by the nature and humans. As a result of human perception through a cultural and societal filter, this objectively present heritage was transformed into a behavioral heritage environment. It therefore started to be noticed in the behavioral world and valued as an important commodity (also economic), worth saving, developing and presenting to the public. Therefore, a reenactment heritage event was created (*Dymarki Swietokrzyskie*) and developed as an important tourist attraction, generating income from heritage and event tourism as well as stimulating the local heritage industry.

**Fig. 5:**
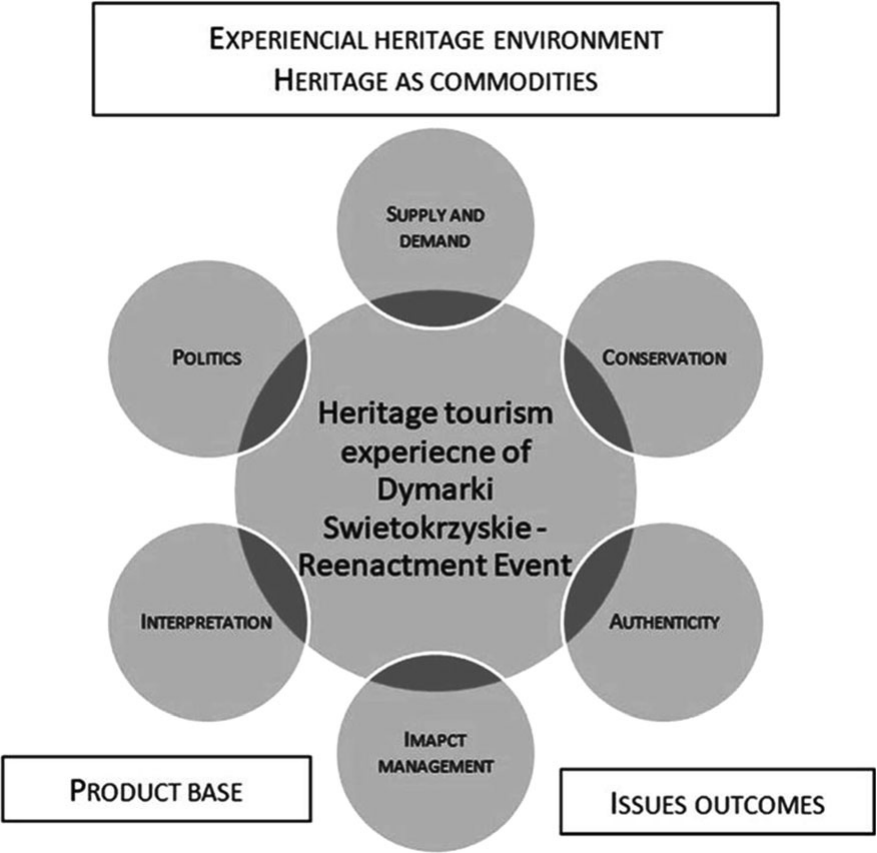
Framework of heritage tourism development at *Dymarki Swietokrzyskie*. *Source*: Own elaboration on the basis of Timothy and Boyd (2003)

According to Timothy & Boyd (2003), creating a heritage tourism experience as the core of heritage tourism is possible due to elements determining its development: politics, supply and demand, conservation, authenticity, impact management, and interpretation. In the case of *Dymarki Swietokrzyskie*, these elements strongly conditioned the creation of the heritage tourism experience (Fig. 5). Regarding politics, it is significant that the research on the ancient metallurgy heritage in the Swietokrzyski region received support from central authorities in the mid-twentieth century. Equally important was support provided in recent years by local authorities (organizational and financial) and NGOs, e.g. an organization of history enthusiasts, reconstruction organizers, and the Polish Tourism and Sightseeing Society.

We must not ignore the importance of the supply and demand issue for the development of heritage tourism based on an event (Fig. 5). In recent decades, both globally (Park, 2013; Timothy & Boyd, 2003) and in Poland (Rohrscheidt, 2008) interest in heritage tourism and heritage events, including reenactments (Carnegie & McCabe, 2008) has been clearly growing and the tourist demand for this kind of attraction has increased. Therefore, the organization of *Dymarki Swietokrzyskie* responds to the growing demand of the contemporary tourism market. It is an element of heritage conservation and as a component of heritage tourism experience development (Timothy & Boyd, 2003). When the event was being created, it was very important to preserve and popularize the ancient metallurgy heritage. At present, it is also highly significant and, as indicated by the organizers, it is one of the reasons it exists.

What is important for developing heritage attractions and the satisfaction tourists derive from them is their authenticity. In the case of *Dymarki Swietokrzyskie* authenticity is maintained thanks to the cooperation with archaeologists, historians, the local Museum of Swietokrzyskie Metallurgy and reenactment enthusiasts from Poland and abroad.

It would not have been possible for the event to function for over 50 years without proper impact management. The organizers are adding new attractions to the event all the time. Subsequent editions included presentations of Roman and barbarian cultures, a local folklore festival, concerts, handcraft and culinary products stands. In this way, a complex tourist product was created, preserving the authenticity of the event. As a result, *Dymarki Swietokrzyskie* is perceived today by visitors and organizers as an important heritage event worth saving and using for local development.

*Dymarki Swietokrzyskie* has an important role in the creation of geographical space of its host town. The event forms an experiential heritage environment and therefore nurtures and influences the creation of heritage tourism in the vicinity of Nowa Słupia. Notably, the event was visited by 19,793 event-goers in 2019. The event organisers emphasized the important role of *Dymarki Swietokrzyskie* in the creation of tourism in the town. Among the respondents of the questionnaire-based survey, the majority of 113 (83.7%) were tourists from outside the town of Nowa Słupia. As Włodarczyk (2009) stated the tourist space is part of geographical space where tourism occurs, therefore the event under study should be treated as a factor creating tourism space in the host town.

The event influences different dimensions of geographical space distinguished by Thrift (2003). *Dymarki Swietokrzyskie* is held at the premises of the Cultural-Archaeological Centre in Nowa Słupia. The centre was built in 2011 as a large facility, co-financed by the EU-funds, and created among others to stage the *Dymarki Swietokrzyskie* event. Therefore, the event is involved in shaping of Thrift’s (2003) first space of empirical constructions in its host town. The event generates tourism movement and thus has its influence on the economy of the town (i.e. creates social and economic flows). As a result of the tourists’ expenditures the accommodation and gastronomic facilities increase profit. The event thus influences the town in unblocking the space of social and economic connections. The image space of the host town is improved by the event. The majority of the respondents of the questionnaire-based survey were very satisfied with the visit and expressed the will to revisit the town. These results may be interpreted as a consequence of a positive host town perception seen by the visitors through the lenses of an interesting event experience. The event forms a place space of Nowa Słupia as well. Specific tourist behavior results from the event, the local heritage resources are involved in the event and transform the place management, tourists', and inhabitants' rhythms of being.

## Conclusion

*Dymarki Swietokrzyskie*, a heritage reenactment event held in a Polish town of Nowa Slupia may be viewed as an event based on the reenactment of past events and ancient industrial traditions of iron smelting. Analysis showed that such an event is a significant tourist attraction. *Dymarki Swietokrzyskie* attracts tourists, is an element of the local heritage industry and contributes to the development of tourism services. It is a part of the heritage tourism sector, developing in Poland and globally.

The analysis confirmed the validity of the Timothy and Boyd’s (2003) heritage tourism behavioral framework. The event is a tourist attraction based on heritage present in the phenomenal environment, which through human perception has been transferred to the behavioral environment and used for creating a contemporary heritage event, evoking specific tourist behaviors and bringing real economic profits. Creating the heritage tourism experience of *Dymarki Swietokrzyskie* was possible due to the influence and convergence of the above-mentioned elements: politics, conservation, authenticity, impact management and interpretation.

This research provides insights into both visitation to and perceptions of an ancient industrial site as part of heritage tourism. This will be valuable both for managers of similar types of tourist attraction sites in terms of management implications and provide a context and methodology for future research. Moreover, the *Dymarki Swietokrzyskie* event has tourism related functions that influences tourism space creation in the town of Nowa Słupia. It also meets all of Thrift’s (2003) dimensions of geographical space due to the experiential heritage environment that the event creates.

The recent pandemic of Covid-19 affected the event sector very badly. A lot of events planned for 2020 have been canceled or postponed like the Tokyo Summer Olympics planned for 2020 and deferred to 2021. In many cases, the host destinations did not achieve the income, and other benefits, that were expected after the realisation of events in 2020. The pandemic impacted visitor perceptions of travel, health infrastructure, safety and crowding related to mass tourism events at destinations (Rowen, 2020; Sato et al., 2020; Zenker & Kock, 2020). However, this year (2021) brought the discovery of effective vaccines against the coronavirus and the development of vaccination programs across different countries. Certainly, in the subsequent years, we will see a new social and economic reality. However, if the vaccine proves to be effective, we will undoubtedly deal with the recovery of the tourism economy after the coronavirus crisis. After overcoming the pandemic crisis destinations will search for opportunities to renew the badly affected tourism and events sector. One way to achieve this goal is the realisation of events, which will certainly be a vital element in rebooting the tourism sector in host areas. After a long period of isolation, people will look for meeting opportunities and new experiences provided by the events sector. Eventually, the years after the pandemic should bring the revival of the event and tourism sector. However, even if the vaccine will prove to be effective, it will be necessary to maintain proper sanitary procedures during events organisation, what will certainly be a challenge for mass event organizers in the post-pandemic period.

Due to the Covid-19 pandemic the *Dymarki Swietokrzyskie event held* in August 2020 was held with strict sanitary procedures (social distancing, wearing masks, etc.). The event was limited to heritage presentations and reenactments and was organised without concerts and other mass gatherings like the Festival of Folk Culture. During the 2020 edition of *Dymarki Swietokrzyskie* 1039 tickets were sold to the visitors attending the event. As we can see the drop in the number of sold tickets, caused by the pandemic, reached 94,8% in relation to 2019.

The event is planned to be organised in a similar way in 2021. *Dynamrki Swietokrzyskie* obviously has a dedicated audience base as reflected by the results of the visitor survey. The event organizers should investigate the use of social media to engage and retain this audience while the physical event is not able to be held on the same scale as before the pandemic. Moving forward in post-pandemic times the event has the advantage of being mostly held outdoors where social distancing will be possible. Moreover, maintaining strict sanitary procedures will be still necessary, because the vaccination process will take time. Also, registration of event-goers, the introduction of tracking applications on their mobile phones, and maybe the introduction of Covid-19 testing or so-called vaccination passports could be necessary in order to avoid the risk of infection during the event. Moreover, the more sustainable-oriented approach to tourism and events will be vital in the post-Covid-19 era. Such an approach could encompass making the events less crowded, more experience-oriented, and more engaging for the participants. Here the role of reenactment heritage events as experience-based products based on strong participants engagement will be very valuable for host areas (Niewiadomski, 2020; Rowen, 2020).

In order to maintain the interest of visitors and to promote the event nation and worldwide the organisers should focus on social media promotion and online streaming. Creating online promotional campaigns, activating bloggers, and presenting the events in social media (like on Facebook or youtube) in real-time will increase the visibility of the event and help address the problems related to the Covid-19 pandemic.
